# Gamma-irradiated copper-based metal organic framework nanocomposites for photocatalytic degradation of water pollutants and disinfection of some pathogenic bacteria and fungi

**DOI:** 10.1186/s12866-024-03587-9

**Published:** 2024-11-06

**Authors:** Gharieb S. El-Sayyad, Ahmed M. El-Khawaga, Huda R. M. Rashdan

**Affiliations:** 1 Drug Microbiology Lab, Drug Radiation Research Department, National Center for Radiation Research and Technology (NCRRT), Egyptian Atomic Energy Authority (EAEA), Cairo, Egypt; 2 Department of Basic Medical Sciences, Faculty of Medicine, Galala University, Galala City 43511, Suez, Egypt; 3 Chemistry of Natural and Microbial Products Department, Pharmaceutical and Drug Industries Research Institute, National Research Centre, 33 El Buhouth St, Dokki, 12622 Giza Egypt

**Keywords:** 1, 2, 3-triazoles, Cu-MOFs, Nanocomposites, Antimicrobial activity, Photocatalytic degradation, Gamma-rays

## Abstract

**Background:**

Although there are many uses for metal–organic framework (MOF) based nanocomposites, research shows that these materials have received a lot of interest in the field of water treatment, namely in the photodegradation of water contaminants, and disinfection of some pathogenic bacteria and fungi. This is brought on by excessive water pollution, a lack of available water, low-quality drinking water, and the emergence of persistent micro-pollutants in water bodies. Photocatalytic methods may be used to remove most water contaminants, and pathogenic microbes, and MOF is an excellent modifying and supporting material for photocatalytic degradation.

**Methods:**

This work involved the fabrication of a unique Cu-MOF based nanocomposite that was exposed to gamma radiation. The nanocomposite was subsequently employed for photocatalytic degradation and as an antimicrobial agent against certain harmful bacteria and fungi. The produced Cu-MOf nanocomposite was identified by XRD, SEM, and EDX. Growth curve analysis, UV lighting impact, and antibiofilm potential have been carried out to check antimicrobial potential. Additionally, the membrane leakage test was used to determine the mechanism of the antimicrobial action. In an experimental investigation of photocatalytic activity, a 50 mL aqueous solution including 10.0 ppm of Rhodamine B (RB) was used to solubilize 10 mg of Cu-MOF. It has been investigated how pH and starting concentration affect RB elimination by Cu-MOF. Ultimately, RB elimination mechanism and kinetic investigations have been carried out.

**Results:**

SEM images from the characterization techniques demonstrated the fact that the Cu-MOF was synthesized effectively and exhibited the Cu-MOF layers' flake-like form. Uneven clusters of rods make up each stratum. The primary peaks in the Cu-MOF's diffraction pattern were found at 2θ values of 8.75^◦^, 14.83^◦^, 17.75^◦^, 21.04^◦^, 22.17^◦^, 23.31^◦^, 25.41^◦^, and 26.38^◦^, according to the XRD data. After 135 min of UV irradiation, only 8% of RB had undergone photolytic destruction. On the other hand, the elimination resulting from adsorption during a 30-min period without light was around 16%. Conversely, after 135 min, Cu-MOF's photocatalytic breakdown of RB with UV light reached 81.3%. At pH 9.0, the greatest removal of RB at equilibrium was found, and when the amount of photocatalyst rose from 5 to 20 mg, the removal efficiency improved as well. The most sensitive organism to the synthesized Cu-MOF, according to antimicrobial data, was *Candida albicans*, with a documented MIC value of 62.5 µg mL^−1^ and antibacterial ZOI as 32.5 mm after 1000 ppm treatment. Cu-MOF also showed the same MIC (62.5 µg mL^−1^) values against *Staphylococcus aureus* and *Escherichia coli*, and 35.0 and 32.0 mm ZOI after 1000 ppm treatment, respectively. Ultimately, it was found that Cu-MOF (1000 µg/mL) after having undergone gamma irradiation (100.0 kGy) was more effective against *S. aureus* (42.5 mm ZOI) and *E. coli* (38.0 mm ZOI).

**Conclusion:**

From the obtained results, the synthesized MOF nanocomposites had promising catalytic degradation of RB dye and high antimicrobial potential which encouraging their use in wastewater treatment against some pathogenic microbes and polluted dyes. Due to the exceptional physicochemical characteristics of MOF nanocomposites, it is possible to create and modify photocatalytic nanocomposites in a way that improves their recovery, efficiency, and recyclability.

## Background

There is an urgent need for research due to the rising threat presented by developing pathogenic fungus species and multidrug-resistant (MDR) strains of bacteria in the context of the increasing worry for global public health [1]. Drug-resistant MDR bacteria use a variety of strategies, and biofilms are a key component of this resistance. The anaerobic environment produced by these biofilms reduces the potency of several antibiotics, including aminoglycosides, fluoroquinolones, and β-lactams [2]. Furthermore, biofilms facilitate the exchange of genes between microbes, which results in the emergence of resistance to antibiotics.

This concerning phenomena has been noted in a variety of geographic locations, posing unexpected risks to human wellness and testing established therapeutic techniques [3]. MDR organisms are more common in clinical settings as antibiotic resistance keeps evolving, which has led to longer hospital admissions, higher rates of morbidity, and higher healthcare expenses [4]. Simultaneously, the advent of fungal infections that are resistant to drugs complicates the treatment of infectious diseases and calls for creative approaches to stop their spread and lessen their negative effects on public health [5].

With their special benefits in combating the expanding problem of multidrug-resistant bacteria and harmful fungi, nanomaterials are emerging as viable substitutes for traditional antimicrobial treatments [6]. In contrast to conventional antibiotics, nanoparticles have a variety of mechanisms of action, including the physical disruption of cell membranes, disruption of vital biological processes, and specific removal of biofilms [7, 8]. Furthermore, nanoparticles show promise as antimicrobial agent delivery vehicles, improving the antimicrobial compounds' stability, solubility, and biocompatibility [9]. With no evidence of resistance creation, nanomaterials provide promising ways to get beyond the restrictions placed on current treatments [10]. The ultimate goal of research is to improve public health by refining and optimizing nanostructures for optimal efficacy against a variety of pathogens [11].

It has been demonstrated that metal-based nanoparticles, such as those made of gold, silver, copper, and selenium, have broad-spectrum antibacterial qualities that can severely impede the development and reproduction of bacteria [12]. The amalgamation of ZnO/CuO antibacterial nanocomposite implanted in chitosan film [13], has been examined in this particular setting. Furthermore, because of its large particular area of the surface, nano-silver that makes a good connection alongside bacteria was used, which greatly slowed down the growth of the germs and accelerated the healing of wounds [14–16]. *Staphylococcus aureus* has been shown to be inhibited by zinc oxide nanoparticles, although calcium and magnesium oxide NPs have potent antibacterial properties with MICs of 6 and 100 mg/L, respectively [17]. ZnO NPs and ZnO-CuO nanocomposites had a positive antibacterial impact on skin infections. [18–21]. Recently, Cu metal is considered as one of the most important and attractive metals in the fabrication of novel MOFs owing to its non-toxic characters, availability, high strength and abundant resources [22–24]. Depending on the kind of nanomaterial and the strain of bacteria under test, these nanomaterials have different MIC and ZOI values [25]. For instance, it has been demonstrated that zinc oxide nanoparticles exhibit MIC concentrations ranging from 0.5 to 8 μg/mL versus *S. aureus* and *E. coli*, whereas Ag NPs possess MIC values varying from 0.5 to 64 μg/mL against a variety of bacterial species [26].

Metal–organic frameworks (MOFs) are strong and extremely porous crystalline solids composed of metal ions or clusters arranged in one, two, or three dimensions, connected by organic molecules known as ligands [27]. Different forms of self-assembly processes, such as solvothermal methods, microwave-assisted technique, sono-chemical method, and electrochemical synthesis, are used to synthesize MOF materials. Zinc, iron, titanium, aluminum, copper, and zirconium are among the most frequently employed metal ions for MOFs synthesis [28]. MOFs are an intriguing family of materials with unique qualities such strong coordination, easy modification, high porosity, high durability towards temperature and chemicals, diversified composition, large surface area, excellent adsorption capacity, and various functions. MOFs and its derivatives are used in many fields, such as energy, storage of gases and separation, remediation of the environment, treatment of water, sensors, purification of air, membrane technology, catalysis, adsorption, and medicine, because of their multi-structural nature, chemical diversity, and unique physicochemical properties [28, 29].

Because of their distinctive qualities, MOFs are a special type of nanomaterials which have generated interest as potential antibacterial agents [28, 30]. The loading capability and release of drugs characteristics of MOFs, which have been demonstrated to have strong bactericidal action and an antibacterial rate that surpasses 99%, may be optimized [31]. MOFs possess distinct advantages over other nanomaterials. These include the capacity to integrate metal ions into their molecular structure, endowing them with inherent antibacterial toxic effects, and the profusion of functional groups that facilitate the modification of MOFs particle exteriors via stealth wrapping and ligand electrochemistry [32, 33].

In this work, metal organic framework nanocomposites based on copper were created and subjected to varying dosages of gamma radiation. Because Cu is chemically inert, MOF composite has greater activity, stability, and safety, which enhances the produced composite's antimicrobial potential. The generated MOF nanocomposite was evaluated for its antimicrobial activity against a range of selected harmful bacteria and fungi in an attempt to counteract antibiotic resistance. This cutting-edge and eco-friendly resource described how nanotechnology may be used to fight pathogenic microbes-induced infections and break down certain contaminated dyes.

## Materials and methods

### Materials

#### Synthesis of the targeting organic linker. N, N'-((sulfonylbis(4,1-phenylene))bis(5-methyl-1H-1,2,3-triazole-1,4-diyl))bis(ethan-1-yl-1-ylidene))bis(hydrazinecarbothioamide) (STHC) (3)

1,1'-((sulfonylbis(4,1-phenylene))bis(5-methyl-1*H*-1,2,3-triazole-1,4-diyl))bis(ethan-1-one) (**1**)(10 mmol) was submitted to react with thiosemicarbazide (**2**) (20 mmol) in the presence of catalytic amount of hydrochloric acid (1 mL) in ethanol (20 mL) under reflux for 5 h. the reaction mixture was cooled and the precipitate was collected and washed with water and recrystallized from acetic acid as white crystals Yield: 71%, m.p.:232-235oC, FT-IR (KBr, cm-1): *v* 3375 (broad, NH, NH_2_), 1610 (C = N), 1560 (C = C); ^1^H-NMR (DMSO-d_*6*_): *δ* 2.40 (s, 6H, 2CH_3_), 2.48 (s, 6H, 2CH_3_), 3.29 (s, 4H, 2NH_2_), 7.84 (d, 4H, Ar–H), 7.25(d, 4H, Ar–H), 10.39 (s, 2H, 2NH); ^13^C-NMR (100 MHz, DMSO-d6): *δ* 9.92 for 2CH_3_, 27.82 for 2 CH_3_, 126.79, 129.54, 138.32, 141.56 and 143.16 for (Ar–C) and 193.45 for C = S;

#### Cu-MOF preparation

Metal–organic frameworks (MOFs) can be considered as new type of microporous crystalline materials constructed via the formation of coordination bonds between the selected metal ions and the organic linkers as shown (Scheme 2) [34, 35].

Cu-MOF was created by gradually adding an aqueous copper acetate solution to an organic linker (1) solution that had been dissolved in 15 ml of DMF and stirred. After that, product was refluxed for 48 h at 80 °C. The solution turned from yellowish orange to a gray precipitate, which was subsequently removed by filtering, cleaned, and vacuum-dried.

#### Photocatalytic activity measurement

In this experiment, a 50 ml aqueous solution including Rhodamine B (RB) at a starting concentration of 10.0 ppm was used to solubilize a total of 10 mg of Cu-MOF. The solubilization procedure was conducted while being constantly stirred. Then, the mixture was put in a light-restricted chamber for 30 min to achieve a condition of equilibrium among the procedures of adsorption and desorption of the photocatalyst and (RB) dye. Following the solution's completion, a UV light is used to irradiate it. To aid in the separation of the photocatalyst, 2.0 ml of the sample was taken out of the tube and centrifuged at regular intervals of 15 min. Using a UV–Visible spectrophotometer, the absorbance of RB solution was determined at a wavelength of 554.0 nm [36]. An equation was used to calculate the percentage of deterioration.

1 $$Percentage\;of\;degradation\;=\frac{Ci\;-\;Cf}{Ci}\;\times\;100$$

Where C_i_ represents the initial absorption of the dye and RB represents its absorption following a different time (min). The identical operation is conducted using variables such as pH, dye concentration and catalyst dose.

#### Tests of in vitro antimicrobial sensitivity (ZOI & MIC)

To obtain a concentration ranging from 62.5—1000 µgmL^−1^, varying quantities of the produced Cu-MOF nanocomposites was separately dispersed in DMSO organic solvent. Samples were then treated with ultrasonic technology. The method of agar well diffusion test was employed to evaluate antimicrobial activity [37]. All of the studied microbial strains' growth constraint was assessed using the zone of inhibition (ZOI). The microbial inoculums were calibrated using a UV–Vis. spectrophotometer at a fixed wavelength of 600 nm, yielding 0.5 McFarland (1–4) × 10^8^ CFU/mL.

For 24 h at 37 °C (for bacteria and unicellular fungi) and 7 days at room temperature (for true fungus), the plates were incubated. At Cairo MIRCEN (Faculty of Agriculture, Ain Shams University, Cairo, Egypt), the antifungal potential of *Alternaria alternate* EUM108 and *Fusarium oxysporum* EUM37, two plant pathogenic fungi, and *Aspergillus brasiliensis* ATCC16404 and *Candida albicans* ATCC10231, two human pathogenic true, and unicellular fungi were assessed. Additionally, only DMSO (negative control) and Nystatin (positive control) were applied to the agar wells concurrently.

In the interim, several pathogenic bacterial strains, such as *Escherichia coli* ATCC11229, *Bacillus subtilis* ATCC6633, *Staphylococcus aureus* ATCC43300, *Klebsiella pneumoniae* ATCC13883, and *Pseudomonas aeruginosa* ATCC6538, were used in the antibacterial susceptibility experiment. To construct control Petri plates, agar wells were treated with DMSO alone (negative control) and amoxicillin/clavulanic acid (positive control).

The inhibitory zones in the agar wells were measured precisely. Agar wells contained the lower concentration of the synthetic Cu-MOF nanocomposites that exhibited the highest degree of inhibition, or the MIC. While MIC is used to evaluate the quantitative measurement as a concentration-dependent activity, ZOI is the most useful criteria for evaluating the qualitative capability as a screening approach for antimicrobial activity [38, 39].

#### Growth curve assay

Growth curve test was used to investigate Cu-MOF's effect on the growth of the most sensitive bacteria, *S. aureus* and *E. coli* as described by Huang et al., [40]. In 5.0 mL nutrient broth tubes, the bacterial culture was fixed to 0.5 McFarland (1 × 10^8^ CFU/mL). Separate equal quantities of Cu-MOF nanocomposites (10 ppm; 10 µg mL^−1^) were added to each of the tubes under examination. Up to 16 h, the absorbance of the growth of bacteria after treatment was measured at intervals of every two hours (wavelength of 600 nm). To generate the normal growth curve, the average of the duplicate measurements was compared to the hourly intervals.

#### Impact of the synthesized MOF nanocomposites on protein leakage

Well-sonicated and distributed Cu-MOF nanocomposites at different concentrations (0.125, 0.25, 0.5, and 1.0 mg/mL) were added to 10 mL of nutrient broth, and 100 µL was injected into the pure 18-h bacterial culture, which was established at 0.5 McFarland (1 × 10^8^ CFU/mL). The control was broth that had been inoculated with culture without any samples. After five hours of incubation at 37°C, all of the treated specimens were centrifuged for fifteen minutes at 5000 rpm [41]. One milliliter of Bradford reagent was mixed with 100 µL of supernatant for each of the samples. The optical density was determined after 10 min of dark incubation at 595 nm [41].

#### Gamma-rays' impact on the antibacterial potential

The freshly created NPs (Cu-MOF) were exposed to gamma-ray illuminating at different doses (25, 50, and 100 kGy) at the NCRRT in Cairo, Egypt. Radiation came from the ^60^Co-Gamma chamber 4000-A in India. The dose rate utilized in this study was 0.892 kGy/h. Gamma irradiation produced solvated electrons and potent free radicals during water radiolysis [42, 43].

#### Impact of UV light on the antibacterial capacity

Bacterial cultures were allowed to develop to a standard 0.5 McFarland (1 × 10^8^ CFU/mL) and then incubated for two hours. Cu-MOF nanocomposites was inserted into the anticipated tubes. The optical density method was used to evaluate the antibacterial efficacy of the synthesized Cu-MOF nanocomposites against *S. aureus* and *E. coli* following UV illumination, as contrasted to the non-UV standard irradiation [44].

There were two sets of tubes used: one set included nanocomposites that had not been subjected to UV radiation, while the other set did. Various exposure durations of 0, 15, 30, 45, 60, and 75 min were applied to the Cu-MOF nanocomposites. The 6.9 mW cm^−2^ was the measurement of the disturbance strength for the samples, which were evaluated at 37 °C. Equation 2 specified the inhibition %, and Abd Elkodous et al., [45], reported that the samples' turbidity was measured at 600 nm.

2 $$Percentage\;of\;bacterial\;growth\;inhibitio\;=\frac{O.\;D\;of\;the\;control\;sample\;-\;O.D\;of\;the\;treated\;sample\;}{O.\;D.\;of\;the\;control\;sample}\times100$$

#### Statistical investigation

The experimental data were expressed using the calculated mean and standard deviation. The calculated mean is derived from triplicate data from three different experiments. With the use of IBM Corp.'s SPSS software, version 22 (New York), one-way analysis of variance (ANOVA) and the least significant difference (LSD) test (0.05 level) were used to examine statistical significance.

## Results and discussion

**Scheme 1:**
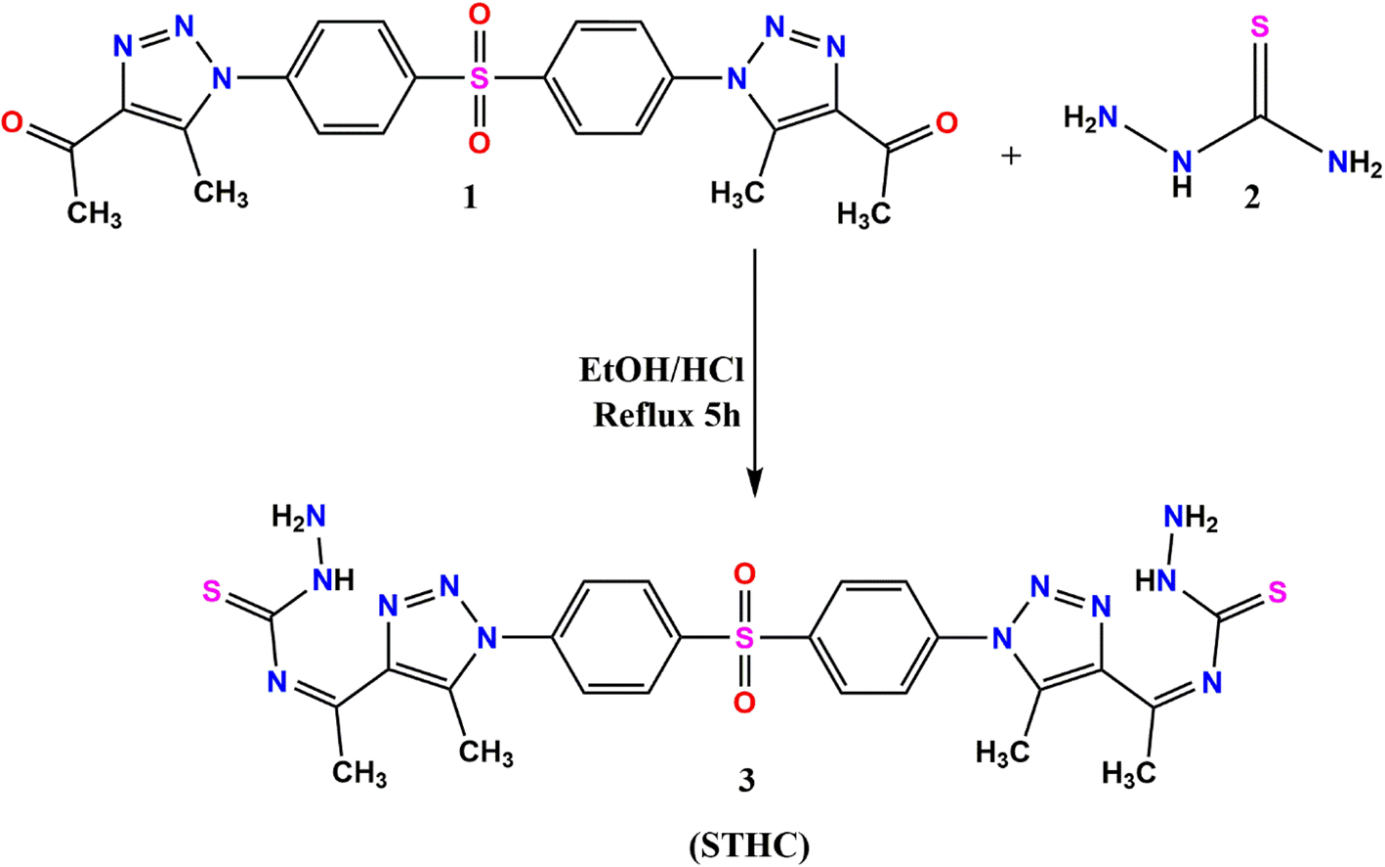
Synthetic procedures of the target molecule STHC

1,1'-((sulfonylbis(4,1-phenylene))bis(5-methyl-1*H*-1,2,3-triazole-1,4-diyl))bis(ethan-1-one) (**1**) [44], was submitted to react with thiosemicarbazide (**2**) in the presence of catalytic amount of hydrochloric acid in ethanol under reflux for 5 h to afford the corresponding hydrazinecarbothioamide derivative *N,N'*-((sulfonylbis(4,1-phenylene))bis(5-methyl-1*H*-1,2,3-triazole-1,4-diyl))bis(ethan-1-yl-1-ylidene))bis(hydrazinecarbothioamide) (STHC) (**3**) (Scheme 1).

**Fig. 1:**
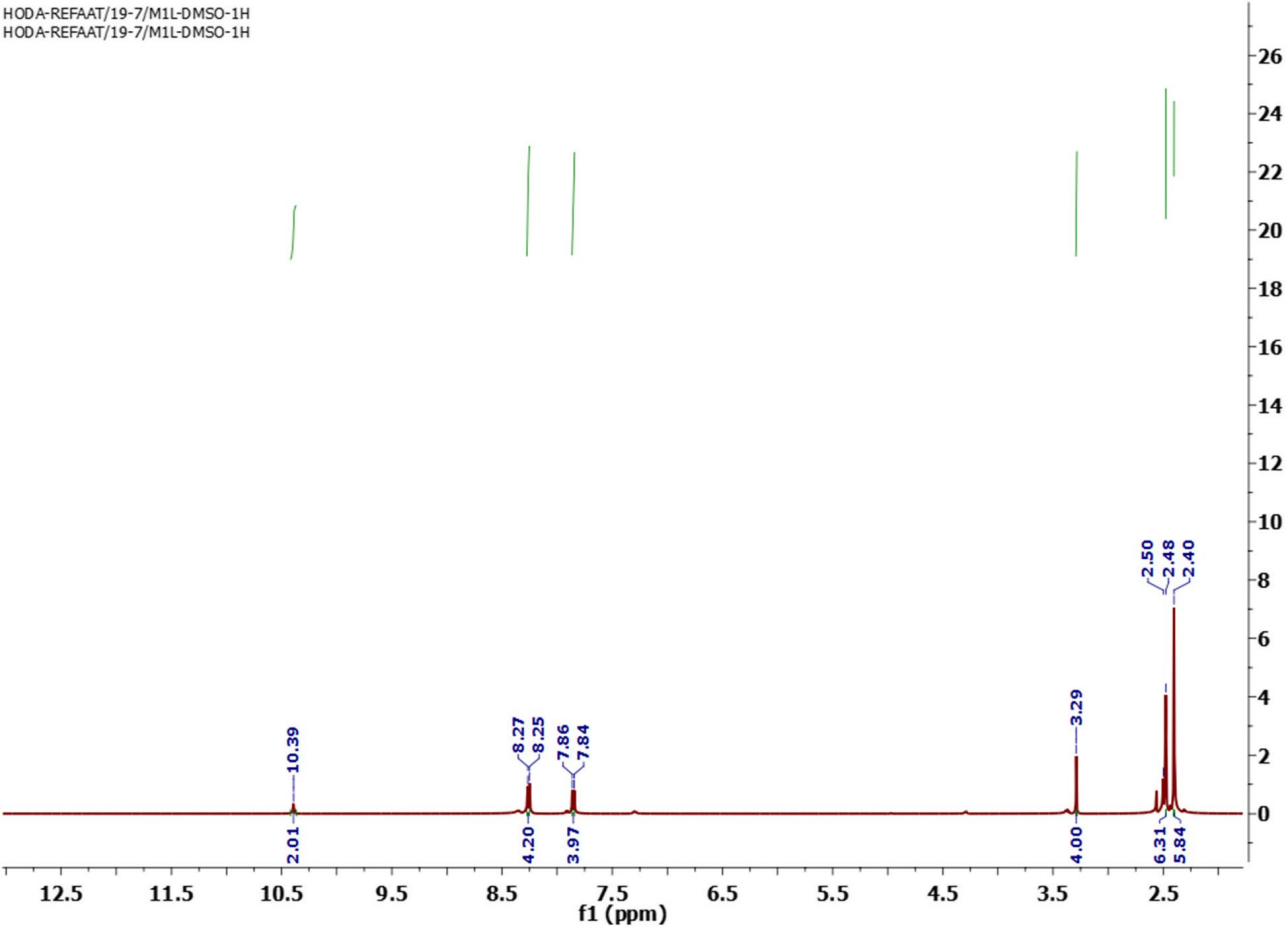
H^1^NMR spectrum of STHC

The chemical structure of STHC was affirmed from correct spectral and micro-analytical data. Its H^1^-NMR spectrum revealed two singlet signals at* δ* 2.40, 2.48 ppm for the 12 protons of the 4 methyl groups. Additionally, its H^1^-NMR spectrum exhibited singlet signals at *δ* 3.29 ppm represented the 4 protons of the two NH_2_ groups and two doublet signals at* δ* 7.84 and 8.25 ppm with coupling constant 10 Hz for the 8 aromatic protons. The protons of the NH groups were appeared as singlet signal at* δ* 10.39 ppm (Fig. 1).

**Fig. 2:**
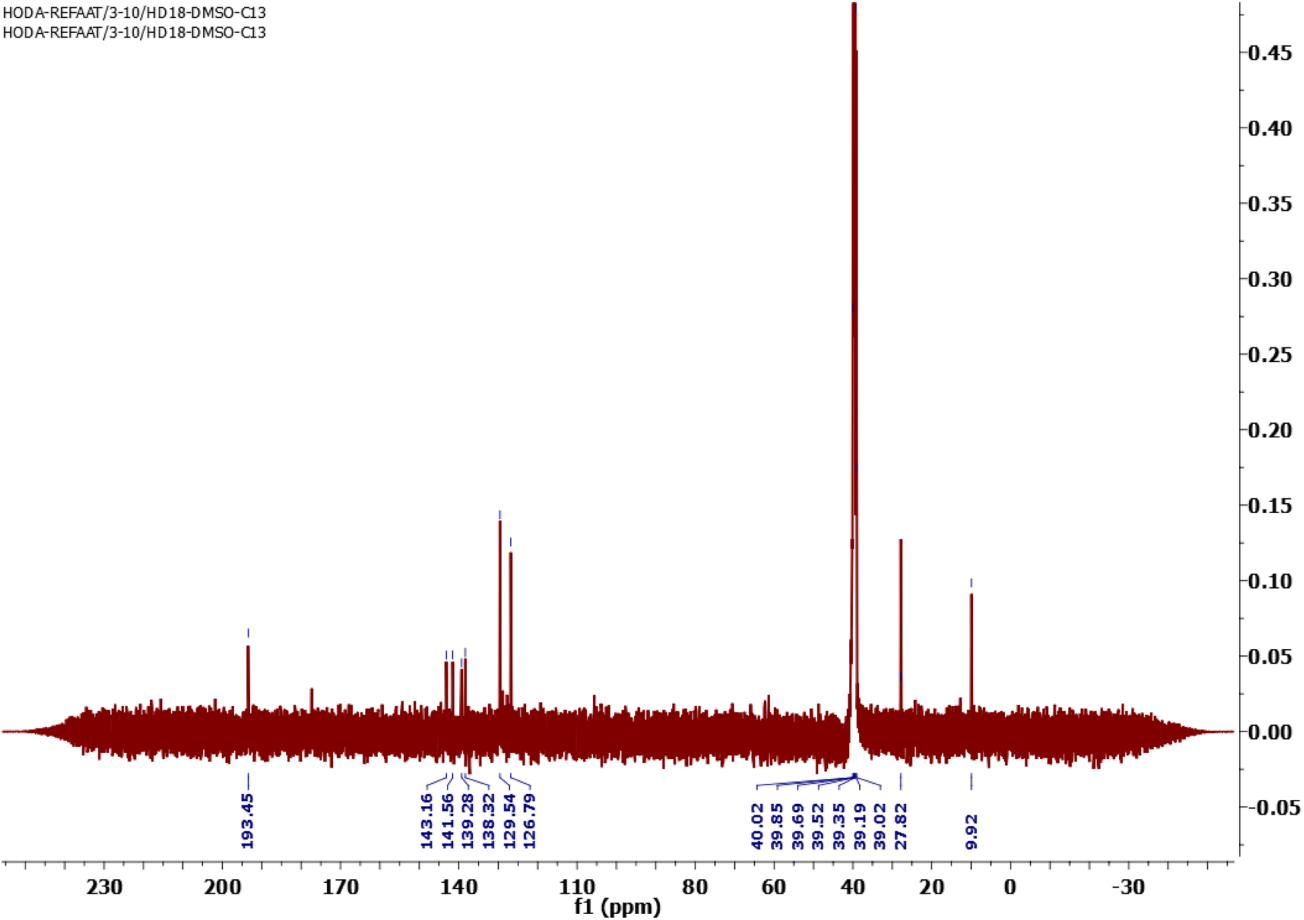
C^13^NMR spectrum of STHC

Moreover, the C^13^-NMR spectrum of STHC showed significant signals at 9.92 for 2CH_3_, 27.82 for 2 CH_3_, 126.79, 129.54, 138.32, 141.56 and 143.16 for (Ar–C) and 193.45 for C = S (Fig. 2).

**Scheme 2:**
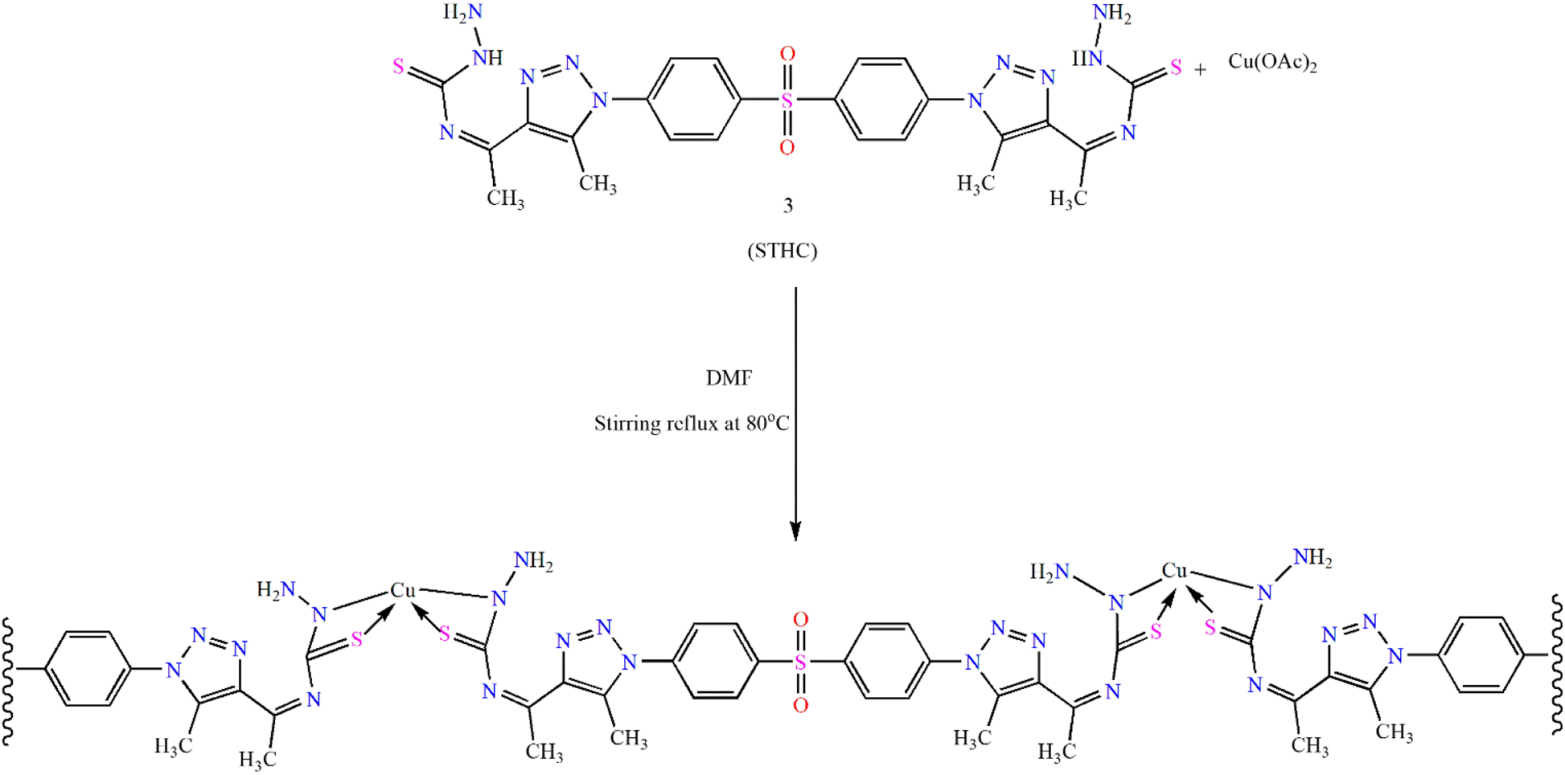
The proposed mechanism of Cu-MOF synthesis

**STHC** was utilized as the organic ligand for the fabrication of the Cu-MOF as shown in (Scheme 2). A light-beige precipitate was collected, washed with ethanol/water, filtered and dried under vacuum.

**Fig. 3:**
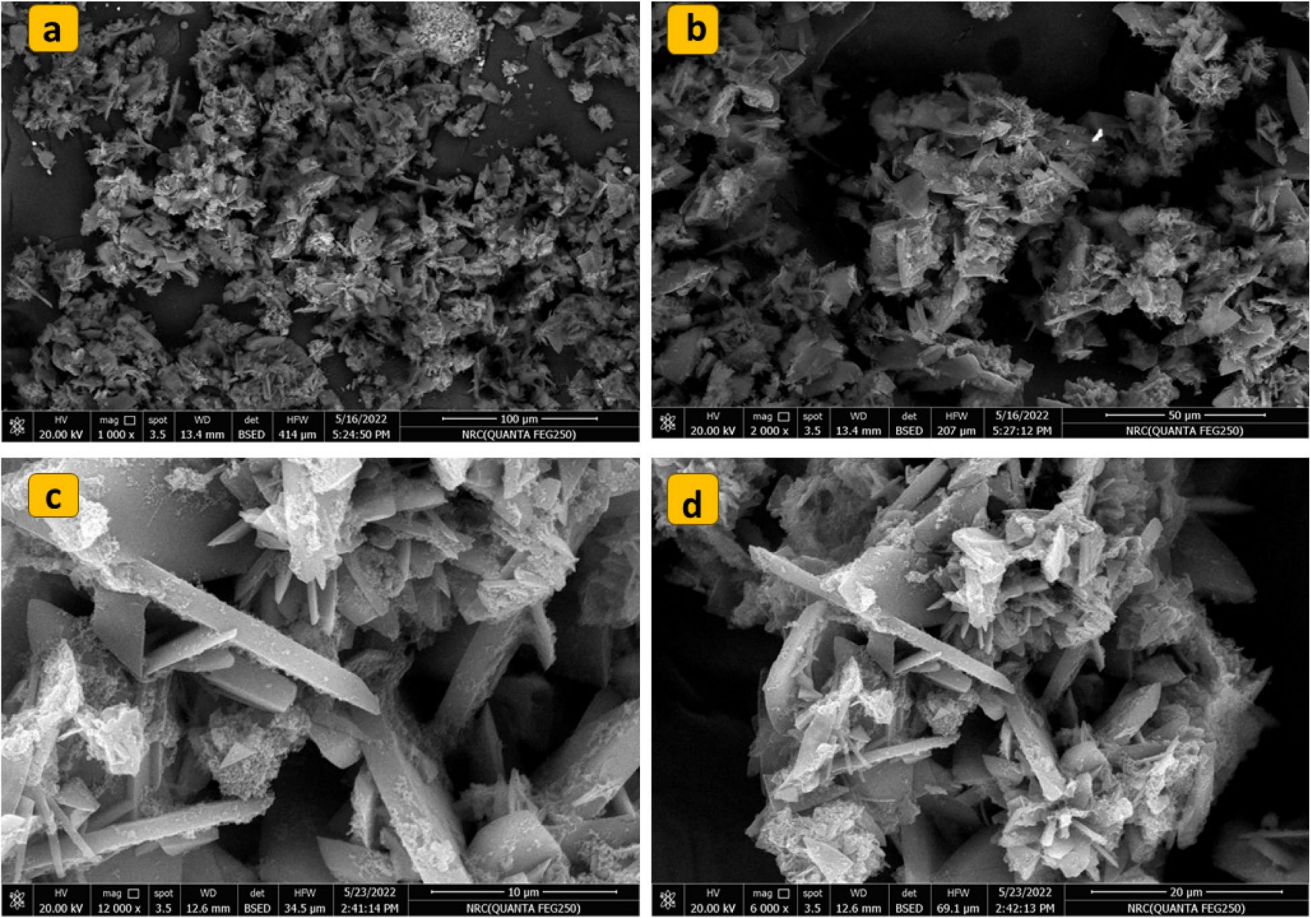
Field-emission scanning electron microscopy images of the Cu-MOF at different magnifications

The morphological structure of the as prepared Cu-MOF was elucidated using The Field-emission scanning electron microscopy (SEM) supported with energy-dispersive X-ray (EDX). The SEM images showed that the Cu-MOF was prepared successfully, they revealed the flake-like shape of the Cu-MOF layers. Each layer consists of irregular aggregates of rods (Fig. 3a-d).

**Fig. 4:**
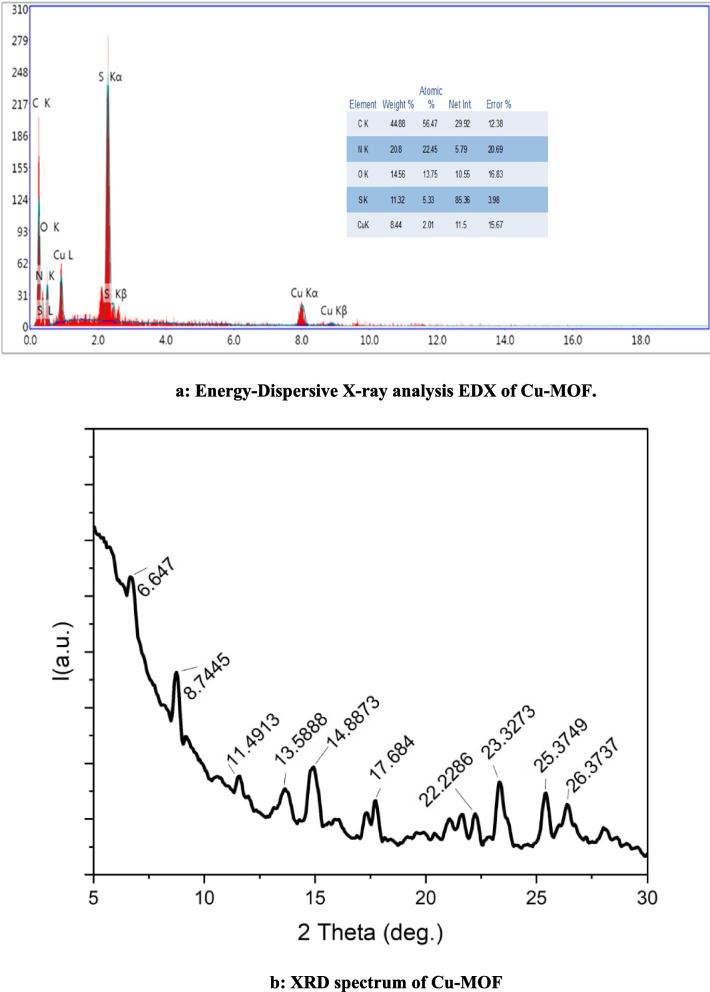
**a** Energy-Dispersive X-ray analysis EDX of Cu-MOF. **b** XRD spectrum of Cu-MOF

The peaks of C, O, N, S, and Cu elements were recorded in the energy dispersive (EDX).

(Fig. 4a). Additionally, the crystal structure and the material composition of the as prepared.

Cu-MOF was further analyzed by X-ray diffraction with a Cu Kα radiation source. The results revealed that the main peaks were observed in the diffraction pattern of the Cu-MOF at 2θ values of 8.75^◦^, 14.83^◦^, 17.75^◦^, 21.04^◦^, 22.17^◦^, 23.31^◦^, 25.41^◦^, 26. 38^◦^.The obtained XRD spectra were compared to the literature [23, 46], and affirmed that all the diffraction patterns were agreed with the Cu-MOF XRD spectrum (Fig. 4b).

Moreover, the apparent highest intense peak residing at ~ 23⁰ is functioned for the determination of the crystallite size. The crystallite size is mathematically expressed according to Scherrer equation as [47–51].

$$D \left(crystallite size in nm\right)=\left[\frac{\kappa .\lambda }{\Delta .Cosine \theta }\right](1x)$$

The parameters in this equation are the shape factor (k), the radiant light wavelength (λ), the full width at the mid-height of the peak (∆) represented in radians, and finally the angle where the diffraction occurs (θ) expressed in radians. Considering these factors, the crystallite size is around 19.7 nm.

### Photocatalytic degradation of Rhodamine B (RB) using Cu- MOF

**Fig. 5:**
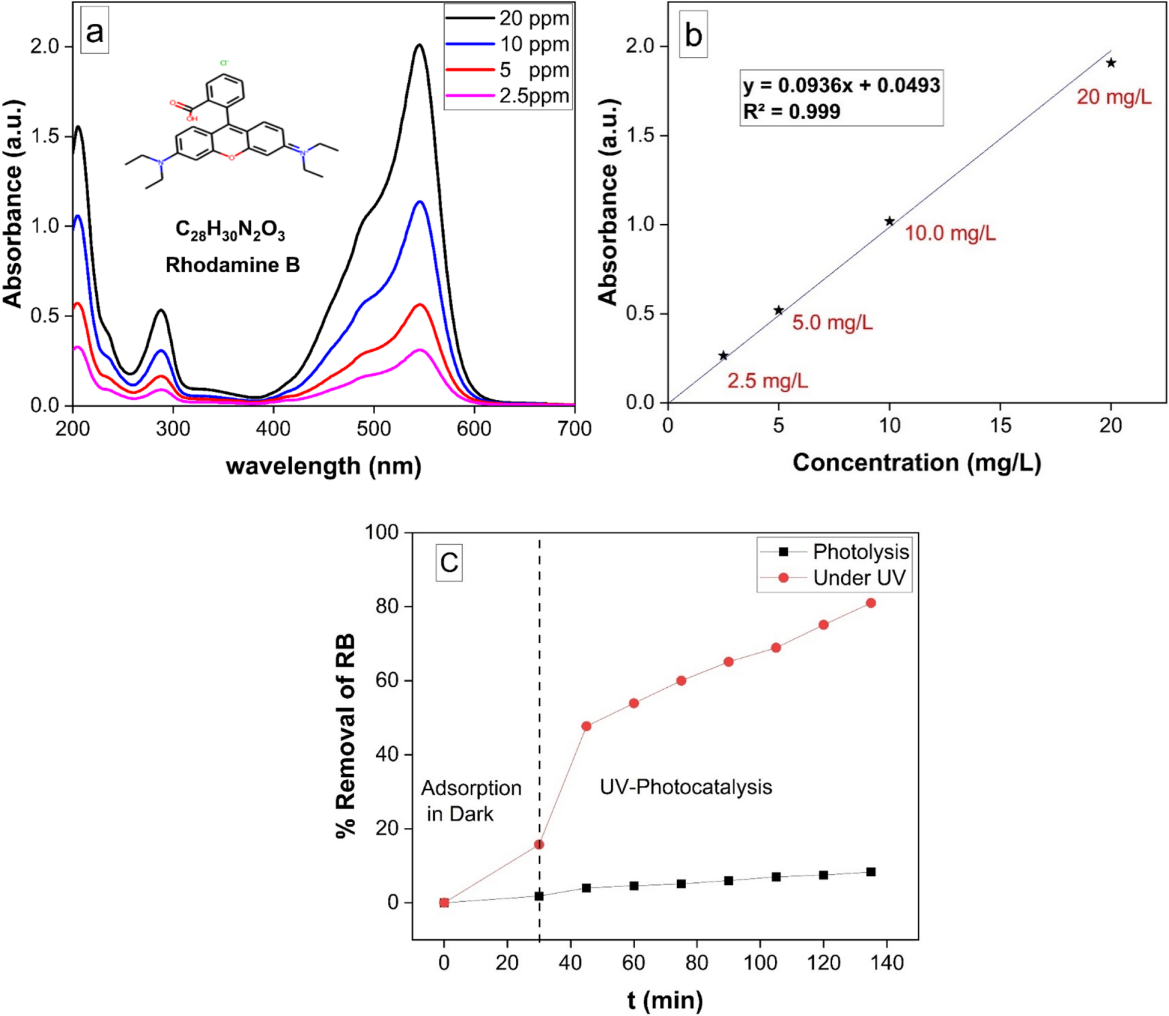
**a** UV-Vis. spectrum of Rhodamine B (RB) at different concentration, **b** calibration curve using (2.5 – 20.0 mg/L) of RB and **c** Removal of RB within 135 minutes due to photolysis without the catalyst (black line), photocatalysis under UV irradiation (Red line).

Using spectrophotometry, the elimination of RB was tracked at the wavelength of maximum absorbance, λmax = 545 nm [52]. The absorption peaks of RB dye at various concentrations ranging from 2.5 to 20 mg/L are clearly seen in Fig. 5A. Figure 5B shows the calibration curve that was utilized for the spectrophotometric examination of RB samples. After 135 min of UV irradiation, as shown in Fig. 5C, only 8% of RB had undergone photolytic destruction. On the other hand, as Fig. 5C illustrates, the elimination resulting from adsorption in dark for 30 min was around 16%. Conversely, after 135 min, Cu-MOF's photocatalytic breakdown of RB pursuant to UV light reached 81.3%. The existence of a metal–semiconductor heterojunction inside a photocatalyst, which facilitates efficient charge separation and improves light absorption, is responsible for this increased photocatalytic efficiency.

### Effect of pH on removal of Rhodamine B (RB) by Cu- MOF

One of the most important factors that must be taken into consideration in dye removal investigations is their dependence on the pH of the solution [53]. In a 135-min experiment with specific parameters (10 mg of Cu-MOF, 50 ml of RB solution (10 mg/L) at 25 °C), we examined the impact of starting pH levels.

**Fig. 6:**
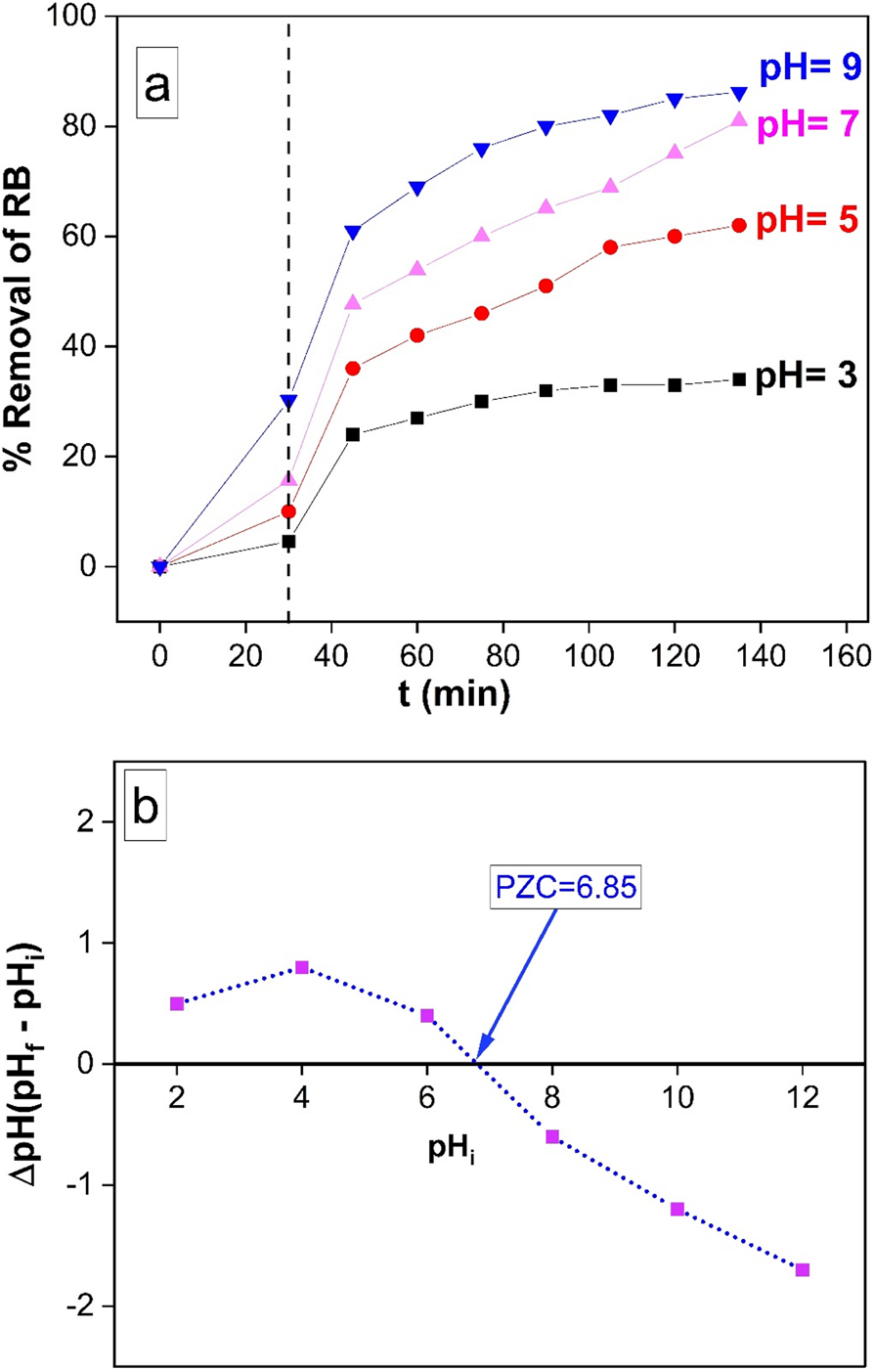
**a** Showing the variation of RB removal (%) with time at different solution pH (3.0, 5.0, 7.0 and 9.0) (10 mg g of Cu-MOF in 50 ml of 10 mg/L RB at 25 ^o^C). **b** Point of zero charge (PZC) of Cu-MOF.

A graphical illustration of the elimination of RB as time passes at various pH values (3–9) is shown in Fig. 6. Notably, as shown in Fig. 6 (A), the highest elimination of RB at equilibrium was found at pH 9.0. Cu-MOF's point of zero charge (PZC) was ascertained by introducing 0.01 g of the compound into 50 mL of a 0.01 M NaCl solution. Using HCl or NaOH, the pH of the solutions was changed to 2, 4, 6, 8, 10, and 12 values. Following the magnetic separation of Cu-MOF, the samples were agitated at 200 rpm for 48 h, and pH readings were taken. The value of the pH at the Point of Zero Charge (PZC) was obtained by graphing the final pH against the beginning pH, as indicated in Fig. 6 (B).

At pH = 6.85, where there was no discernible change between the end and initial pH readings, the PZC pH was found. According to this discovery, the Cu-MOF photocatalyst's surface charge is positive when the pH is under the PZC threshold and negative when the pH is higher. Additionally, the photocatalyst's surface charge becomes neutral at the PZC pH, resulting in less electrostatic interaction with ions like RB ions. Cu-MOF's negative net surface charge at pH 9.0 is responsible for the efficient photocatalytic breakdown of RB. This negative charge accelerates the degradation process by attracting the positively charged RB and the negatively charged Cu-MOF. On the other hand, around pH 5.0, as Cu-MOF's net surface charge increases, the degradation of RB starts to slow down. This causes repulsion forces to form between the positively charged surface of Cu-MOF and the positively charged surface of RB [54].

### Effect of initial concentration of Rhodamine B (RB) and Cu- MOF dose on Rhodamine B (RB) degradation efficiency

**Fig. 7:**
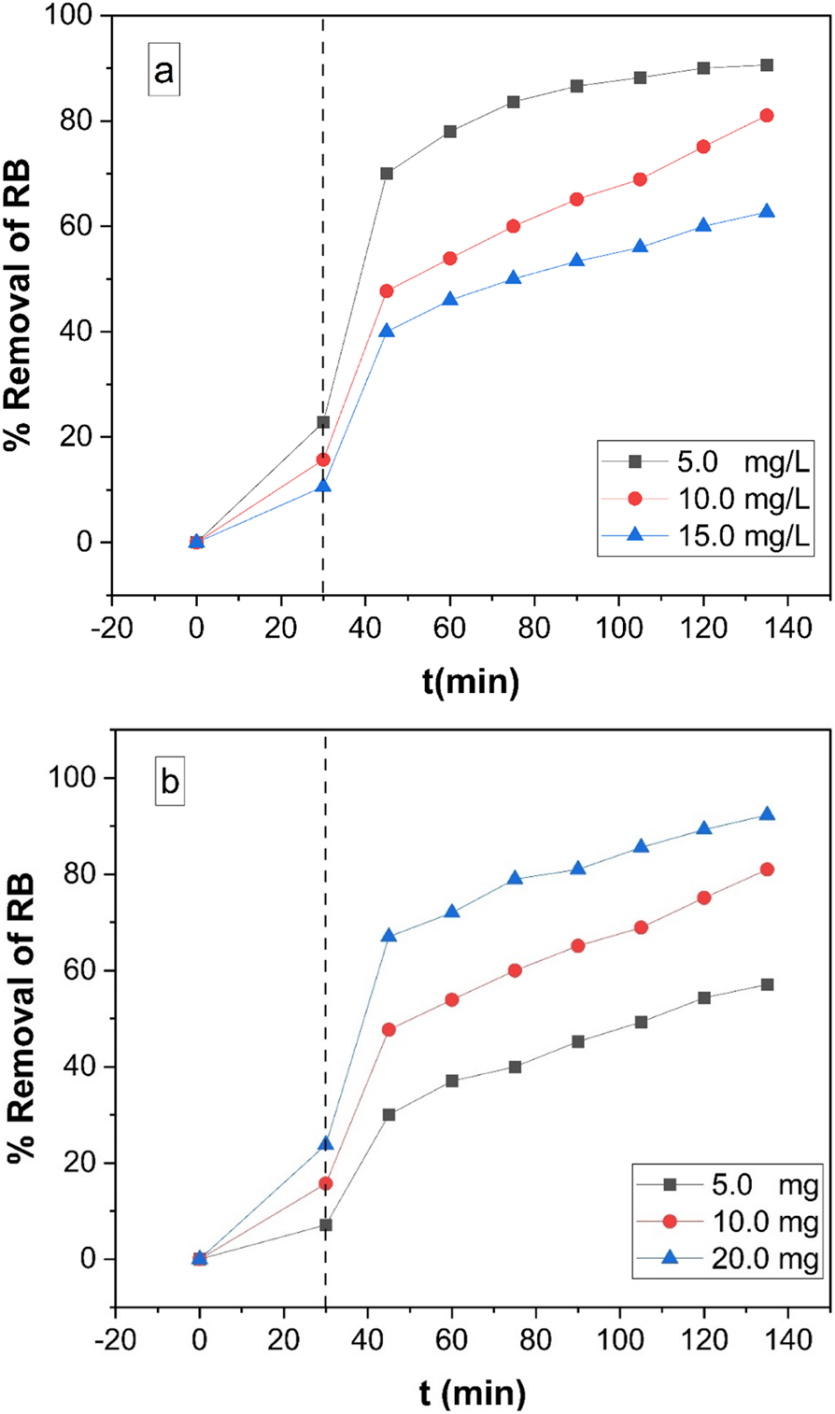
**a **The variation of percent removal as a function of contact time at different initial RB concentrations (5.0 – 15.0 mg/L) at pH 9 and 10.0 mg Cu-MOF and **b** Effect of the photocatalyst dose on the removal efficiency of RB (50 ml RB solution (10 mg/L), Temp. = 25 ^o^C and pH 9).

We looked at how changing the starting RB concentration might affect Cu-MOF's ability to remove it while keeping the reaction circumstances unchanged. The change in the percentage of RB elimination over time for various starting RB concentrations (5.0 – 15.0 mg/L) is shown in Fig. 7(A). The findings show that the clearance efficiency rose when the initial RB concentration dropped from 5 to 15 mg/L. This implies that once faced with UV light in the presence of the synthetic photocatalyst, RB may be efficiently eliminated, even at low starting concentrations. This occurred because less light was able to pass through the solution and reach the Cu-MOF surface as a result of the dye's initial concentration increasing and the solution's concentration of unabsorbed dye increasing. As shown in Fig. 7(B), the impact of different dosages of Cu-MOF on the effectiveness of RB elimination using UV light was investigated by altering the photocatalyst's amount from 5 to 20 mg while maintaining the RB concentration at 10 mg/L. The outcomes demonstrated that when the dosage of photocatalyst was raised from 5 to 20 mg, the removal efficiency rose as well. The expanded active area or active sites of the photocatalyst in relation to the volume ratio of the RB solution may be the cause of the improvement in removal efficiency that occurs with increasing photocatalyst amount in the reaction [53].

### Kinetic studies

The following formula (Eq. 3) can be used to calculate the rate of RB elimination:

3 $$-\;\ln\;Ct\;/CO\;=\;-\;Kt$$

*Where*, C_t_ and C_o_ denote the remaining and initial concentrations of RB, respectively, while t represents the removal time, and k represents the removal rate constant.

**Fig. 8:**
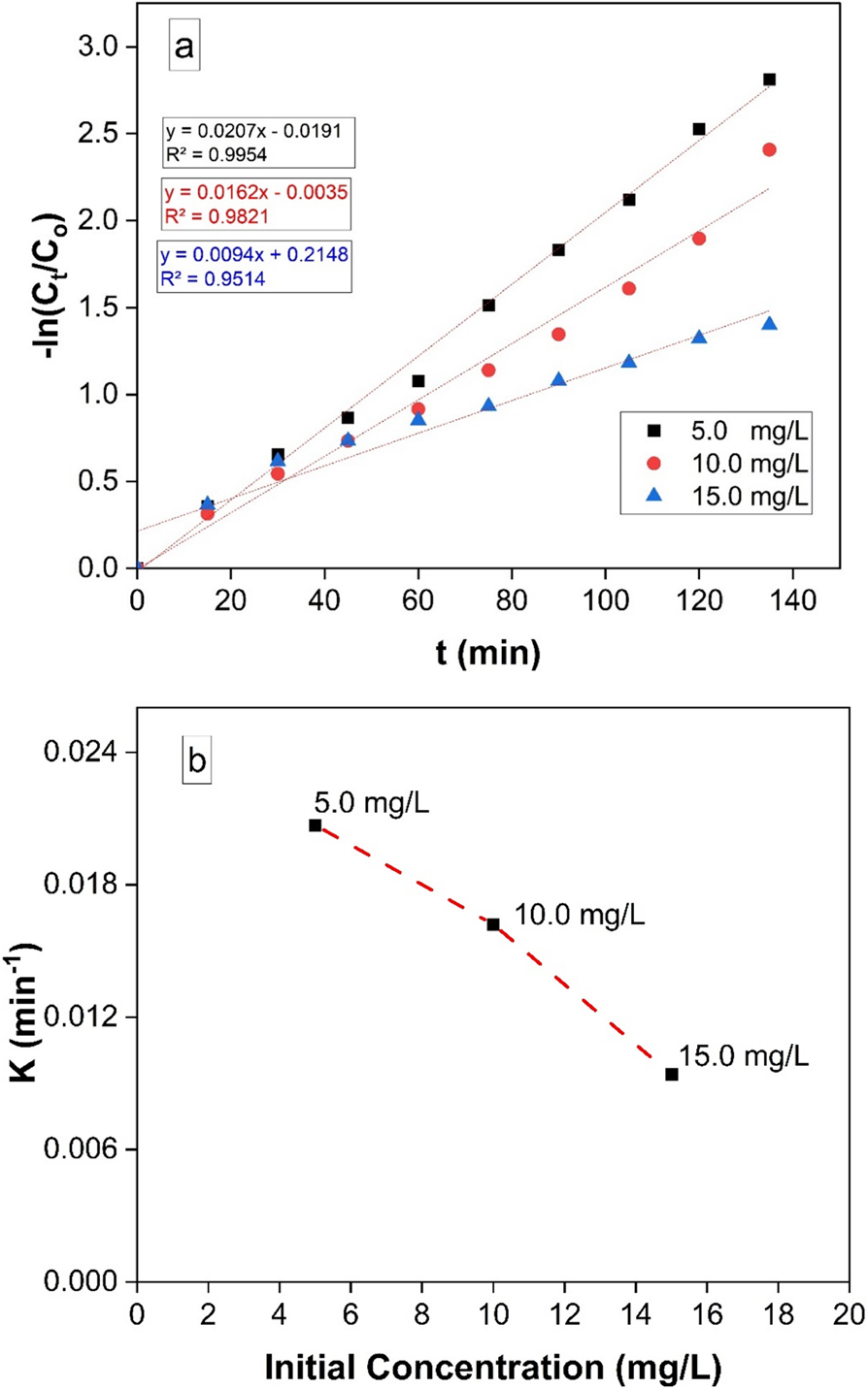
**a** Kinetics plots for linear fitting of data obtained from pseudo-first-order reaction model for RB degradation under UV light irradiation and 10 mg catalyst, 50 mL of 5, 10, and 15 mg /L dye concentration, and **b** Shows a relation of apparent pseudo-first-order rate constants vs. initial concentration of RB.

The association among -ln (Ct/Co) and t is seen in Fig. 8(A). The results showed that the reaction's removal kinetics followed pseudo-first-order rate kinetics. As can be shown in Fig. 8 (B)**,** arise in the initial concentration of RB caused the apparent pseudo-first-order rate constants to rise. According to earlier research, there is a link among reaction rate constants and RB concentration [55].

### Mechanism of photocatalysis of Rhodamine B (RB)

The following can be used to clarify the possible mechanism: Variations in pH levels impact the processes of photodegradation, which include the involvement of hydroxyl radicals, oxidation through holes that are positive in the valence band, and reduction by electrons in the conduction band. When UV light encourages the production of pairs of electrons and holes on the surface of Cu-MOF, photocatalytic degradation is predicted to occur in the presence of Cu-MOF [56, 57]. Because of their oxidative potential, these holes can either oxidize the reactive RB to form a degradation product or react with -OH groups to make hydroxyl radicals. The interactions among RB and the used photocatalyst are summed up in Eqs. (4–5).4


5


(Or)


6



7


**Fig. 9:**
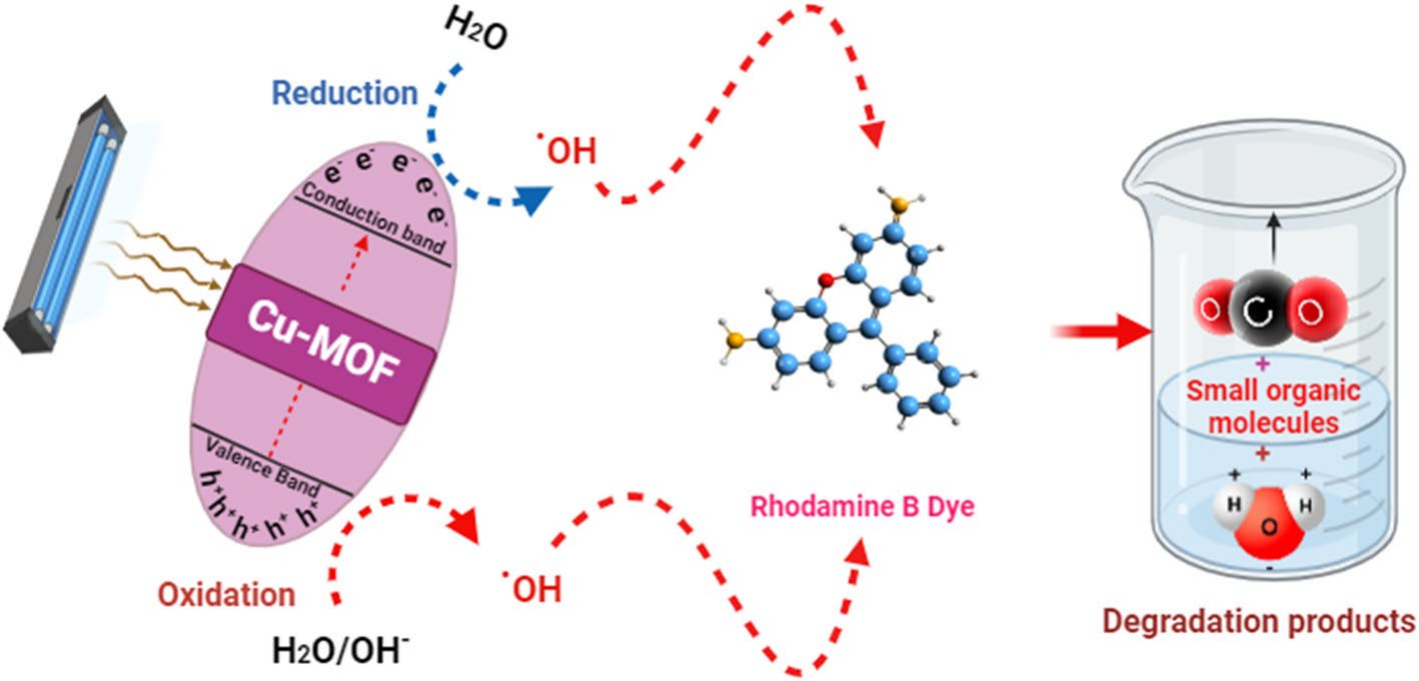
Proposed mechanism of photocatalytic degradation of RB by Cu-MOF nanocomposites

The proposed mechanism of connection among the generated Cu-MOF and RB is shown in Fig. 9. Cu-MOF are excited by UV light, which results in the production of charge carriers that start redox processes.

Consequently, smaller organic compounds are formed as a result of the breakdown of RB by the ensuing free radicals, such as OH^·^ and O_2_^·−^. It is notable that a few publications about the byproducts of degradation of RB have been published as of this writing. Thus, additional research using analytical methods like gas chromatography-mass spectrometry (GC–MS) and high-performance liquid chromatography (HPLC) is necessary for a more thorough examination of the breakdown products of RB.

### Antimicrobial activity (ZOI & MIC)

Table 1 displays the antifungal properties of the produced Cu-MOF nanocomposites against two human pathogenic fungi and two plant-harming fungi. The synthesized Cu-MOF nanocomposites showed promising antifungal potential when compared to the traditional antifungal, nystatin. The information gathered (Table 1) further shown that the fungal pathogen under investigation affects the MIC-values of the produced Cu-MOF nanocomposites. Data showed that *C. albicans* was the unicelluar fungi and the most sensitive to the produced Cu-MOF nanocomposites; 32.5 mm ZOI at 1000 ppm and recorded MIC readings for Cu-MOF nanocomposites were 62.5 µg mL^−1^ (Table 1).

**Table 1:** Antifungal activity of Cu-MOF against different human and plant pathogenic fungi

Sample concentration		Diameter of inhibition zone (mm)			
**(µg mL**^**−1**^**)**		***C. albicans***	***A. brasiliensis***	***A. alternata***	***F. oxysporum***
**Cu-MOF**	0.00 (C)	0.00^f^	0.00^d^	0.00^e^	0.00^c^
	62.5	8.00 ± 0.50^e^	0.00^d^	0.00^e^	0.00^c^
	125	14.0 ± 1.00^d^	0.00^d^	9.00 ± 1.50^d^	0.00^c^
	250	22.5 ± 0.52^c^	10.00 ± 0.55^c^	12.30 ± 1.00^c^	0.00^c^
	500	28.0 ± 1.00^b^	14.00 ± 1.00^b^	14.00 ± 0.50^b^	13.50 ± 1.00^b^
	1000	32.5 ± 0.55^a^	15.50 ± 0.50^a^	18.50 ± 1.50^a^	16.00 ± 1.50^a^
**Nystatin**		12.00 ± 0.50	9.00 ± 0.55	8.50 ± 0.50	0

Nystatin was used at a concentration of 100 µg mL^−1^. Calculated mean is for triplicate measurements from three independent experiments ± SD, ^a−f^ means with different superscripts in the same column for each nanocomposites are considered statistically different (LSD test, P ≤ 0.05)

Table 2 displays the antibacterial characteristics of the produced Cu-MOF nanocomposites against five distinct human pathogenic bacterial strains. The data acquired amply revealed the wide range of antibacterial properties of the produced Cu-MOF nanocomposites when contrasted with the standard antibacterial agent (Amoxicillin/Clavulanic acid). The most resistant bacteria were *P. aeruginosa* and *K. pneumoniae*, although the Cu-MOF sample was most efficient against *S. aureus* and *E. coli*.

**Table 2:** Antibacterial activity of Cu-MOF against different Gram-positive and Gram-negative human pathogenic bacterial strains

Sample concentration		Diameter of inhibition zone (mm)				
**(µg mL**^**−1**^**)**		***E. coli***	***S. aureus***	***P. aeruginosa***	***K. pneumoniae***	***B. subtilis***
**Cu-MOF**	0.00 (C)	0.00^f^	0.00^f^	0.00^d^	0.00^d^	0.00^e^
	62.5	12.00 ± 1.50^e^	14.00 ± 1.50^e^	0.00^d^	0.00^d^	0.00^e^
	125	18.00 ± 1.00^d^	20.60 ± 1.00^d^	0.00^d^	0.00^d^	11.50 ± 1.50^d^
	250	22.50 ± 0.50^c^	28.50 ± 1.50^c^	12.50 ± 0.50^c^	10.50 ± 0.50^c^	16.50 ± 1.00^c^
	500	29.50 ± 0.55^b^	33.30 ± 1.00^b^	16.00 ± 1.90^b^	12.00 ± 1.00^b^	19.00 ± 1.50^b^
	1000	32.00 ± 1.0^a^	35.60 ± 0.50^a^	21.60 ± 1.50^a^	14.50 ± 0.55^a^	20.50 ± 0.50^a^
**Amoxicillin/Clavulanic acid**		10.00 ± 0.15	14.00 ± 0.55	9.50 ± 1.50	9.00 ± 0.55	10.00 ± 1.50

Amoxicillin/Clavulanic acid was used at a concentration of 100µg mL^−1^. Calculated mean is for triplicate measurements from three independent experiments ± SD, ^a−f^ means with different superscripts in the same column for each nanocomposites are considered statistically different (LSD test, P ≤ 0.05)

The data obtained also demonstrated that the stated minimum inhibitory concentrations (MIC) for Cu-MOF nanocomposites were 62.5 µg mL^−1^ toward *S. aureus* and *E. coli*, and 35.0 and 32.0 mm ZOI at 1000 ppm, similarly (Table 2). Numerous studies have examined MOF as effective antibacterial agents for a range of illnesses [38, 58–61].

When considering the low percentage of metal ions that assists in reducing the toxic degree of the produced Cu-MOF nanocomposites and the particular arrangement between Cu and metals in the MOF nodes that enhanced characteristics enable for the possible use in different fields of medicine with the appropriate treatment, the combined potential of Cu metal and the design of the MOF in the produced Cu-MOF nanocomposites accounts for the most significant antimicrobial activity at low concentrations [62–64].

Rising resistance to antibiotics is a critical global health concern that requires the development of new antimicrobial formulations in order to treat drug-resistant microorganisms. Recently, there has been a lot of interest in the research of microbial resistance to drugs in treatments that use NPs as antimicrobial agents [65]. Further tests were carried out to evaluate the efficacy of synthesized MOF materials produced in this study as potential antimicrobials against a variety of multi-drug-resistant strains of bacteria in addition to many hazardous fungi that affect humans and plants.

### Bacterial growth curve examination

**Fig. 10:**
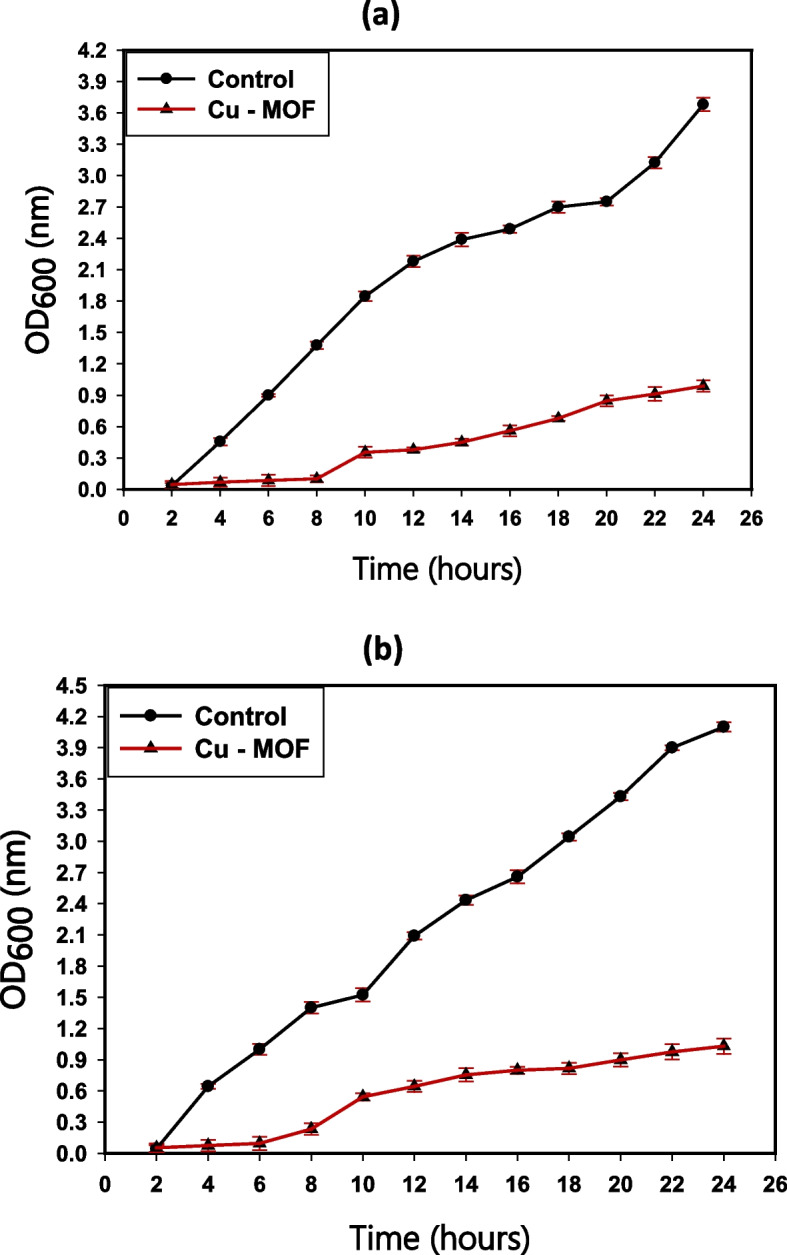
The effect of Cu-MOF nanocomposites on the growth curve of (**a**) *S. aureus*, and (**b**) *E. coli*

The kinetic analysis findings of the synthetic MOF nanocomposites  (62.5 µg/mL) on the development and propagation of the tested *E. coli* and *S. aureus* are displayed in Fig. 10. The growth dynamics of the untreated control *S. aureus* were normal, and the O.D. value at a specific wavelength (600 nm) was 3.68 nm (Fig. 10a). After adding Cu-MOF nanocomposites, there was a positive noticeable impact on the kinetic growth curve. The determined O.D. was observed at 0.99 nm (Fig. 10a), indicating a potential suppressive impact on the kinetics of *S. aureus* growth. Furthermore, the growth dynamics of the untreated control *E. coli* were normal, and the O.D. value at a specific wavelength (600 nm) was 4.09 nm (Fig. 10b). After adding Cu-MOF nanocomposites, there was a positive noticeable impact on the kinetic growth curve. The determined O.D. was observed at 1.03 nm **(**Fig. 10b), indicating the future inhibiting influence on the kinetics of *E. coli* growth. Bacterial cells may die as a result of the generated samples' surface producing reactive oxygen species (ROS) [66, 67]. The distinct ROS generated by Cu-MOF nanocomposites, that also results in oxidation of proteins, bacterial DNA destruction, and lipid peroxidation, has the ability to harm the germs under investigation. Furthermore, the production of positive ions such as copper ions increases the lethal connection with the Cu-MOF and the bacteria's membrane since *S. aureus* and *E. coli* cells contain a negative charge.

Because of their nanoscale, equilibrium, utmost purity, charge on the surface, and chemical arrangement, the created Cu-MOF nanocomposites had a favorable reactivity that might improve the possibility of interacting with more harmful bacteria. It is noteworthy that the antibacterial activity findings, as determined by ZOI and growth curve tests, were in agreement and demonstrated that Cu-MOF nanocomposites, even at low doses (62.5 µg/mL), had an inhibitory effect on the studied bacterial growth kinetics.

### Bacterial protein leakage examination

**Fig. 11:**
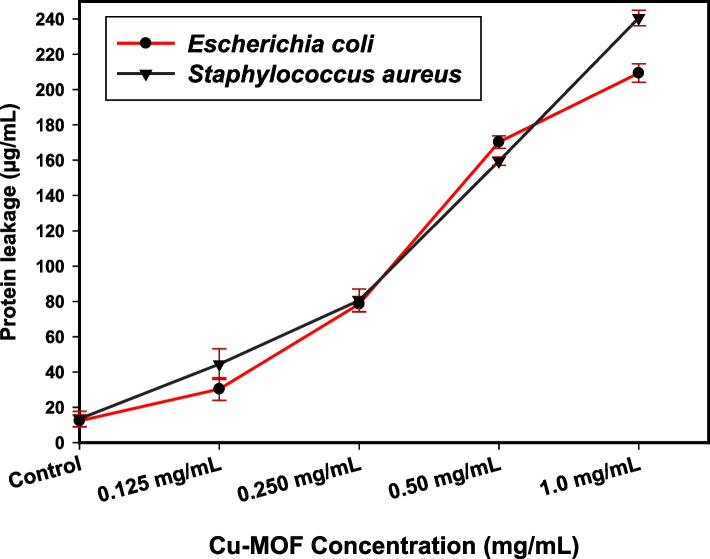
The effect of Cu-MOF nanocomposites on the protein leakage from *S. aureus*, and *E. coli* cell membranes

The Bradford technique was used to calculate the quantities of proteins released from the treated suspensions of *E. coli* and *S. aureus* [68]. According to Fig. 11, the amount of protein removed from *S. aureus* and *E. coli* is directly proportional to the concentration of Cu-MOF nanocomposites (at varying concentrations). After treatment with Cu-MOF nanocomposites (1.0 mg/mL), the amount of protein removed is counted to be 240.5 µg/mL and 209.3 µg/mL, respectively. This indicates the antibacterial properties of Cu-MOF nanocomposites and explains the appearance of holes in the membranes of *S. aureus* and *E. coli*, which aid in the process of the proteins leaking out of the bacterial cytoplasm.

The results showed that raising the quantity of Cu-MOF nanocomposites improved the permeability of membranes directly correlated with the amount of bacterial protein eliminated. The primary factor responsible for the inhibition of the bacterial biomass is the favorable impact on the permeability of the membrane with respect to the leakage of proteins.

When coupled NPs, corresponding investigations like [69] and [70] describe comparable outcomes that indicate concentration-dependence for bacterial membrane dislodgement and possible leaking of intracellular bacterial organelles to the extracellular structure of cells.

### Gamma-rays' impact on antibacterial activity

The generated Cu-MOF nanocomposites was subjected to gamma doses of 25.0, 50.0, and 100.0 kGy, and the antibacterial activity of the compound was evaluated. Cu-MOF nanocomposites (1000 µg/mL) gamma-irradiated (100.0 kGy) was more effective against *S*. *aureus* (42.5 mm ZOI) and *E*. *coli* (38.0 mm ZOI), as Table 3 demonstrates. Recent studies [71–73], suggest that the increased antibacterial effectiveness against all tested microbial strains  may be due to the reduced crystallite size of the synthesized nanocomposites following gamma irradiation.

**Table 3:** Antibacterial potential of Cu-MOF nanocomposites as zone of inhibition (ZOI; mm), after irradiation at different gamma-ray doses

**Pathogenic microbes**	**Cu-MOF nanocomposites (1000 ppm)**			
	**Control** **(non-irradiated)**	**25 kGy**	**50 kGy**	**100 kGy**
*S. aureus*	32.5 ± 0.55	38.0 ± 1.50	40.5 ± 0.50	42.5 ± 1.00
*E. coli*	29.0 ± 1.00	31.0 ± 1.55	33.5 ± 1.00	38.0 ± 0.50

### Effect of UV-rays on the antibacterial activity

**Fig. 12:**
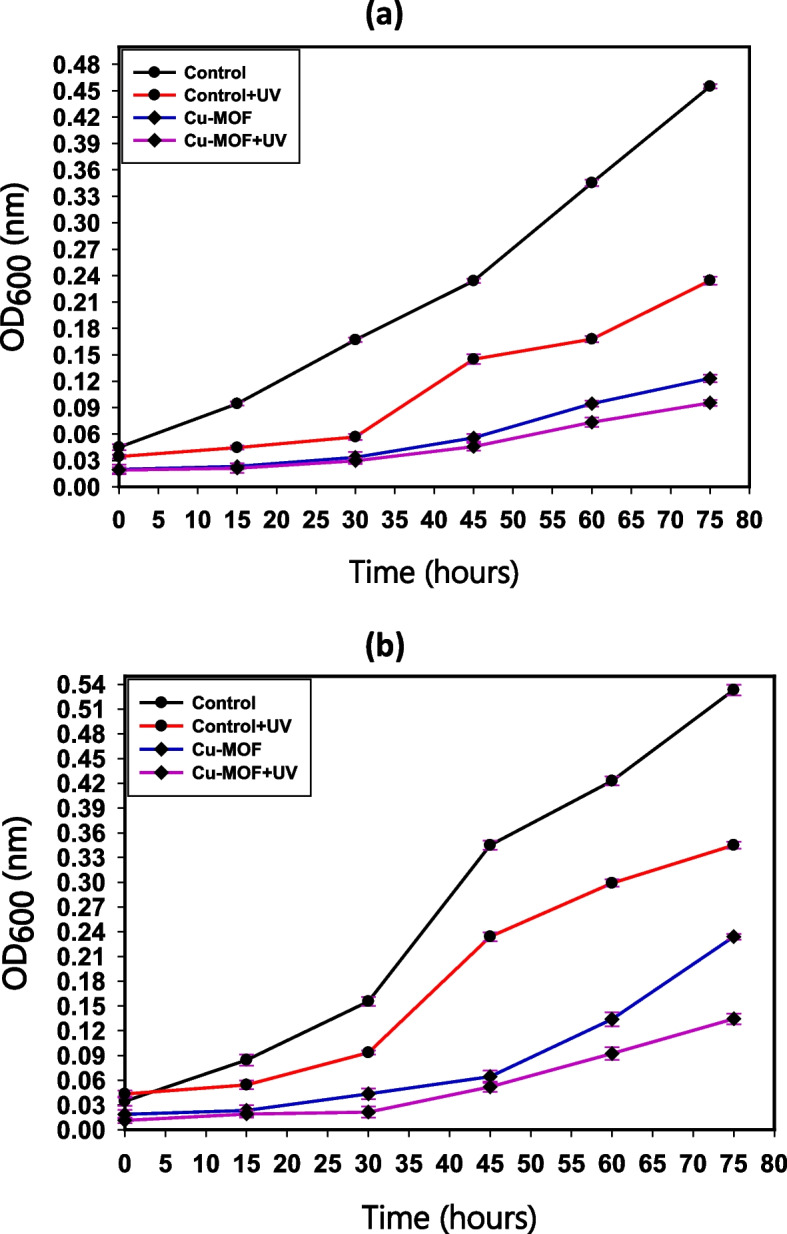
The UV effect on the antibacterial activity of Cu-MOF nanocomposites against* S. aureus* (**a**),and* E. coli *(**b**)

Figures 12a and b, respectively, demonstrate how *S. aureus* and *E. coli* may be rendered inactive by UV radiation; the degree of sensitivity increases with exposure time. Positive effects were seen on *S. aureus* and *E. coli* adherence and proliferation over the whole length of the display (0 to 90 min with 15 min time increments). Cu-MOF nanocomposites treatment led to a considerable suppression of *S. aureus* and *E. coli* growth in comparison to the untreated comparative research. The UV experiment's bacterial growth reached its lowest point due to the breakdown that follows UV radiation. It was found that UV light exposure would increase the likelihood that Cu-MOF nanocomposites would be photo-activated.

Cu NPs in Cu-MOF nanocomposites absorb photons and play a role in the creation of freshly formed ROS (O_2_^−^ and H_2_O_2_) in addition to catalytic hydroxyl (OH^.^) when O_2_ and H_2_O exist (in the atmospheric and/or water states). Once stimulated by UV radiation, Cu-MOF nanocomposites serves as a very efficient disinfectant [74]. Microbial decontamination can be conceptualized as ROS (H_2_O_2_) reacting with membranes. The effective barrier was seen once the microorganisms had infiltrated, and the oxidative hydroxyl free radicals continued to be stable and active [67].

### Proposed reaction mechanism of the produced Cu-MOF nanocomposites against Pathogenic *bacteria*

Based on the MOF nanocomposites' antibacterial activity results, we conducted further investigation on the mechanism of action of the produced MOF nanocomposites (Cu-MOF) on the bacterial cell.

**Fig. 13:**
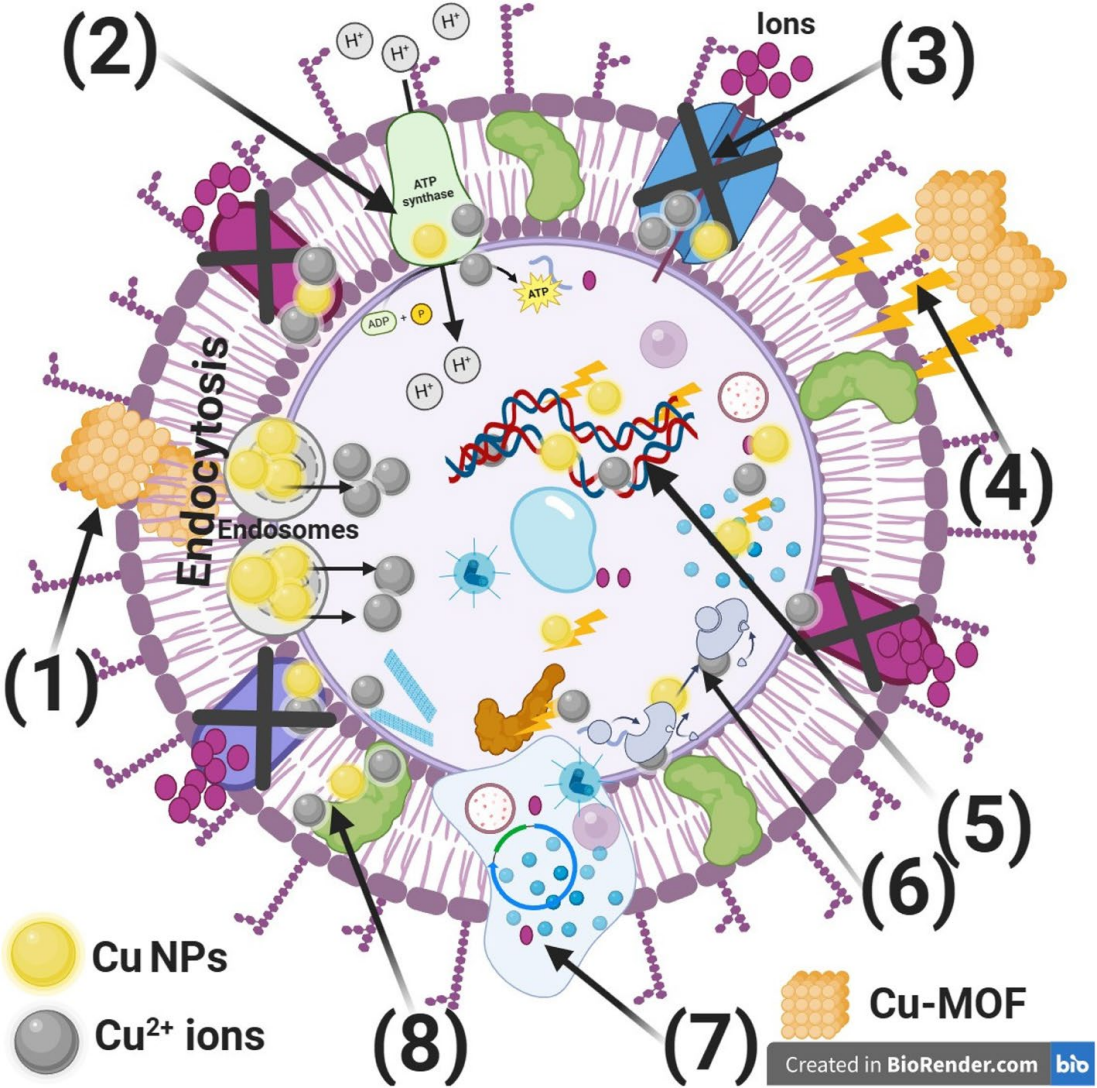
Presents the predicted mechanism of reaction of the produced Cu-MOF nanocomposites toward the bacterial cell. These reactions include the following: **1**) Cu-MOF nanocomposites adheres to the bacterial cell's exterior and causes membrane failure, endocytosis, the formation of endosomes, and changed transport potential, **2**) Cu-MOF nanocomposites harm the electron transport chain; **3**) Cu-MOF nanocomposites prevents ions from passing through the bacterial cell, **4**) Cu-MOF nanocomposites produces and increases ROS, suggesting that the wall of the bacterial cell is beginning to weaken, **5**) Cu-MOF nanocomposites enters bacterial cells and interacts with organelles (such as DNA) to change how those components operate and cause lysis of the cells, (**6**) Cu-MOF nanocomposites interacts with metabolism and enzymes, (**7**) Cu-MOF nanocomposites disrupts the cell membrane, causing internal organelles to seep out, and (**8**) Cu-MOF nanocomposites inhibits the membrane protein. In the cytoplasm and layer, where the existence of a proton motive force could lead the pH to drop below 3.0 and induce the discharge of copper ions, MOF nanocomposite might also function as a carrier to effectively release copper ions. The figure was designed by BioRender.com

It is unclear how precisely Cu-MOF nanocomposites impact bacterial cells. The several methods that affect the action of Cu-MOF nanocomposites are illustrated in Fig. 13. These processes include adherence to bacteria's cell wall and membrane, disruption of internal organelles and biomolecules during cell penetration, production of oxidative stress, and modification of signaling cascades [32, 75].

Because of their ability to damage bacterial cells by adhering to essential cellular structural components, especially their SH groups, the generated Cu-MOF nanocomposites are believed to have bactericidal properties [76]. Moreover, they generate reactive oxygen species and free radicals, which damage cell membranes and obstruct respiratory enzymes [77].

Because the produced Cu-MOF nanocomposites produce free radicals, which damage membranes, they are believed to have antibacterial qualities [78]. The produced Cu-MOF nanocomposites have the ability to halt bacterial cell division and replication, which would result in bacterial cell death. Copper ions interact strongly with thiol groups present in phosphorus-containing bases and enzymes [78].

## Conclusion, future work and limitation

Using a reduction-precipitation method, copper was synthesized after MOF was created using hydrothermal synthesis in the present study. SEM, XRD, and EDX methods were used to characterize the produced MOF samples. The agar well diffusion test technique was utilized to measure antimicrobial properties such as ZOI, and MIC against certain chosen harmful bacteria and fungi. The synthesized MOF compound showed promising antimicrobial potential when compared to traditional antifungal and antibacterial drugs. The produced Cu-MOF nanocomposites was tested for its potential antibacterial action (ZOI) following gamma doses of 25.0%, 50.0, and 100.0 kGy. Beneficially, Cu-MOF nanocomposites exposed to 100 kGy gamma radiation exhibited greater activity versus *S. aureus* (42.5 mm ZOI) and *E. coli* (38.0 mm ZOI). The outcomes of a kinetic analysis that assessed the impact of the synthetic samples at 10 µg/mL on the development and proliferation of the examined *S. aureus*. The growth dynamics of the untreated control *S. aureus* were normal, and the measured O.D. value at a specific wavelength (600 nm) was 3.68 nm. After adding Cu-MOF nanocomposites, a positive impact on the kinetic growth curve was seen, and the determined O.D. was found at 0.99 nm, indicating the potential inhibiting effect on the kinetics of *S. aureus* development. The membrane leakage assay shows the antibacterial properties of Cu-MOF nanocomposites and describes the formation of holes in the *S. aureus* and *E. coli* membrane, which aid in causing the proteins to bleed out of the bacterial cytoplasm. The amount of *S. aureus* and *E. coli* protein eliminated is directly correlated following raising the amount of Cu-MOF nanocomposites (at various concentrations) and measured to be 240.5 µg/mL and 209.3 µg/mL, respectively, resulting from the treatment with Cu-MOF nanocomposites (1.0 mg/mL). After 135 min of UV irradiation, only 8% of RB had undergone photolytic destruction. On the other hand, the elimination resulting from adsorption during a 30-min period without light was around 16%. After 135 min, Cu-MOF's photocatalytic breakdown of RB with UV light reached 81.3%. At pH 9.0. These findings provide fresh hope for promising therapies by combining MOF with Cu to address the issues of water pollution and microbial resistance, particularly in biomedical and environmental applications. In our future work, we may be able to determine the antimicrobial reaction mechanism by measuring DNA damage (the MOF and groove interaction). Finally, in order to verify the degree of Cu oxidation in the MOF material, XPS analysis is necessary.

## Data Availability

The datasets used and/or analyzed during the current study are available from the corresponding author (Gharieb S. El-Sayyad) on reasonable request.
